# Organ Preservation in Rectal Adenocarcinoma: a phase II randomized controlled trial evaluating 3-year disease-free survival in patients with locally advanced rectal cancer treated with chemoradiation plus induction or consolidation chemotherapy, and total mesorectal excision or nonoperative management

**DOI:** 10.1186/s12885-015-1632-z

**Published:** 2015-10-23

**Authors:** J. Joshua Smith, Oliver S. Chow, Marc J. Gollub, Garrett M. Nash, Larissa K. Temple, Martin R. Weiser, José G. Guillem, Philip B. Paty, Karin Avila, Julio Garcia-Aguilar, on behalf of the Rectal Cancer Consortium

**Affiliations:** 1 Memorial Sloan Kettering Cancer Center, 1275 York Avenue, SR-201, New York, NY 10065 USA; 2 Sloan Kettering Institute, 1275 York Avenue, SR-201, New York, NY 10065 USA

**Keywords:** Rectal cancer, Non-operative management, Chemoradiation therapy, Total mesorectal excision. Total neoadjuvant therapy, Clinical complete response, Pathologic complete response

## Abstract

**Background:**

Treatment of patients with non-metastatic, locally advanced rectal cancer (LARC) includes pre-operative chemoradiation, total mesorectal excision (TME) and post-operative adjuvant chemotherapy. This trimodality treatment provides local tumor control in most patients; but almost one-third ultimately die from distant metastasis. Most survivors experience significant impairment in quality of life (QoL), due primarily to removal of the rectum. A current challenge lies in identifying patients who could safely undergo rectal preservation without sacrificing survival benefit and QoL.

**Methods/Design:**

This multi-institutional, phase II study investigates the efficacy of total neoadjuvant therapy (TNT) and selective non-operative management (NOM) in LARC. Patients with MRI-staged Stage II or III rectal cancer amenable to TME will be randomized to receive FOLFOX/CAPEOX: a) before induction neoadjuvant chemotherapy (INCT); or b) after consolidation neoadjuvant chemotherapy (CNCT), with 5-FU or capecitabine-based chemoradiation. Patients in both arms will be re-staged after completing all neoadjuvant therapy. Those with residual tumor at the primary site will undergo TME. Patients with clinical complete response (cCR) will receive non-operative management (NOM). NOM patients will be followed every 3 months for 2 years, and every 6 months thereafter. TME patients will be followed according to NCCN guidelines. All will be followed for at least 5 years from the date of surgery or—in patients treated with NOM—the last day of treatment.

**Discussion:**

The studies published thus far on the safety of NOM in LARC have compared survival between select groups of patients with a cCR after NOM, to patients with a pathologic complete response (pCR) after TME. The current study compares 3-year disease-free survival (DFS) in an entire population of patients with LARC, including those with cCR and those with pCR. We will compare the two arms of the study with respect to organ preservation at 3 years, treatment compliance, adverse events and surgical complications. We will measure QoL in both groups. We will analyze molecular indications that may lead to more individually tailored treatments in the future. This will be the first NOM trial utilizing a regression schema for response assessment in a prospective fashion.

**Trial registration:**

NCT02008656

## Background

Standard treatment of patients with non-metastatic, locally advanced rectal cancer (LARC) includes preoperative chemoradiation (CRT), total mesorectal excision (TME) and postoperative adjuvant chemotherapy (ACT) [1]. This intensive tri-modal therapy provides local control of disease in a majority of patients, and has been adopted as the standard of care in the United States. However, nearly one-third of patients die from distant metastasis (DM) [2], which presumably develops from micrometastases that are clinically occult at the time of surgery. Furthermore, most survivors experience functional sequelae due to removal of the rectum, which significantly impairs their quality of life (QoL) [3]. Thus, current challenges in the treatment of LARC are twofold: 1) Prolongation of survival by reducing the risk of DM; 2) Preservation of QoL in surviving patients by safely avoiding rectal resection.

ACT in LARC patients consists of 4 months of 5-Fluorouracil (5-FU), Leucovorin and Oxaliplatin (FOLFOX), or Capecitabine and Oxaliplatin (CapeOx), starting within 8 weeks after surgery. Unfortunately, more than one-third of eligible patients never start CT because they develop post-operative complications, are too debilitated following surgery, or simply refuse it. Furthermore, less than half of eligible patients receive the full course of FOLFOX [4,5]. It has been proposed that delivery of all CT before rather than after surgery may increase treatment compliance [6,7]. Through early initiation of optimal systemic CT, this approach may enhance the effectiveness of CT in eradicating micrometastasis, and ultimately improve survival. Delivery of systemic chemotherapy before surgery has other potential benefits, such as improving primary tumor response and reducing the interval to diverting loop ileostomy (DLI) takedown. Our institution has experience with delivery of CT at systemic doses before TME, either before or after CRT, in patients with LARC. We recently reported excellent safety profile and treatment compliance in a series of 32 LARC patients treated with FOLFOX before CRT. [8] All patients who underwent TME had an R0 resection, and nearly half had a tumor response greater than 90 %, including 30 % who had either a pathologic complete response (pCR) or a clinical complete response (cCR). The Timing of Rectal Cancer Response to Chemoradiation Trial (TIMING trial, NIH NCT00335816), which completed accrual in 2012, showed that delivering 2, 4, or 6 cycles of FOLFOX after CRT—a concept known as consolidation chemotherapy (CNCT)—in LARC patients increased pCR rates to 25 %, 30 %, and 38 %, respectively, compared to CRT alone (18 %), without an associated increase in adverse events or surgical complications. Notably, 80 % of patients received CNCT without interruption [9]. These data suggest that the use of both CRT and CT before surgery--a concept known as total neoadjuvant therapy (TNT)—improves compliance with systemic CT when compared to postoperative ACT, and may increase tumor response.

A proportion of patients with LARC have a pCR to CRT. It is widely acknowledged that patients with a pCR have lower rates of tumor recurrence, and improved survival, compared to those who do not [10,11]. This leads us to question whether there is a durable benefit to removing the rectum in patients with a pCR. Curing the cancer without removing the rectum could potentially decrease long-term functional problems, and preserve QoL [12].

This randomized phase II trial is designed to test the hypothesis that patients with LARC treated with TNT and TME, or NOM, will have improved 3-year DFS compared to patients treated with CRT, TME and ACT. This study investigates the efficacy of two neoadjuvant chemotherapy (NCT) schedules combined with CRT, in an effort to maximize the proportion of patients with LARC who may be cured without undergoing radical surgery. Patients with LARC who are considered candidates for a low anterior resection, a coloanal anastomosis (CAA) or abdominoperineal excision (APE) will be randomized to receive NCT, either a) before (Induction arm) or b) after (Consolidation arm) CRT. Patients will be restaged 8 (+/- 4) weeks after completing all neoadjuvant therapy. Those with incomplete tumor response will undergo TME. Patients with cCR will be observed, and only those showing signs of tumor relapse during follow-up will undergo TME. We have also put forth a novel regression schema to improve the uniformity of response assessment that will be tested and validated in a prospective fashion. To add value, we will measure QoL in patients treated with TNT and TME in comparison to patients treated with TNT alone. Additionally, we will compare induction chemotherapy (INCT) to consolidation chemotherapy (CNCT) with respect to organ preservation at 3 years, treatment compliance, adverse events and surgical complications.

## Methods/design

### Study design

**Fig. 1:**
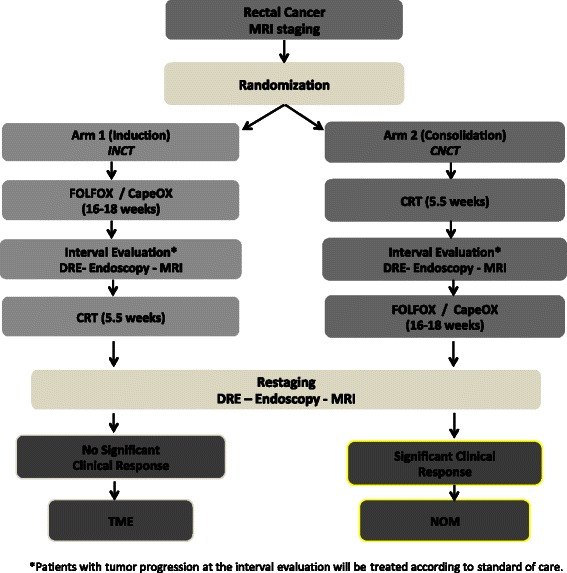
Trial schema. MSKCC-based multi-institutional, Phase II trial schema underway to test the feasibility of incorporating a NOM approach to the multimodality treatment of rectal cancer. This study will evaluate the 3-year DFS in LARC patients treated with CRT plus induction or consolidation chemotherapy and TME or NOM (https://clinicaltrials.gov/ct2/show/NCT02008656?term=NCT02008656&rank=1)

This is a two-arm, randomized, multi-institutional phase II study investigating the efficacy of TNT and selective NOM in LARC patients. Patients with MRI-staged T2-3, N0 or T-any, N1,2 rectal cancer amenable to TME will be randomized to receive FOLFOX/CapeOx before or after 5-FU or capecitabine-based CRT. The study schema is presented in Fig. 1.

### Study objectives

The primary objective of the study is to evaluate 3-year recurrence-free survival (RFS) in patients managed with INCT or CNCT, CRT and selective NOM, compared with standard historical controls managed with CRT and TME followed by CT. Secondarily, this study seeks to: a) compare outcomes between patients in the two study arms with respect to rates of organ preservation, compliance with the neoadjuvant protocol, and adverse events; and b) measure patient-reported functional outcomes and QoL in patients with LARC treated with NCT, CRT and NOM, and compare these to patients treated with TME.

Correlative studies will be completed to: a) investigate the diagnostic performance of conventional and diffusion-weighted magnetic resonance imaging (DW-MRI) in identifying patients with LARC who are treated with NCT and CRT, who may benefit from NOM; b) evaluate the feasibility of using circulating tumor DNA and micro-RNA (miRNA) profiles in plasma to monitor tumor response; c) profile rectal cancers treated with NCT and CRT using next-generation sequencing; and d) investigate molecular mechanisms of tumor resistance to neoadjuvant therapy.

### Trial organization

**Fig. 2:**
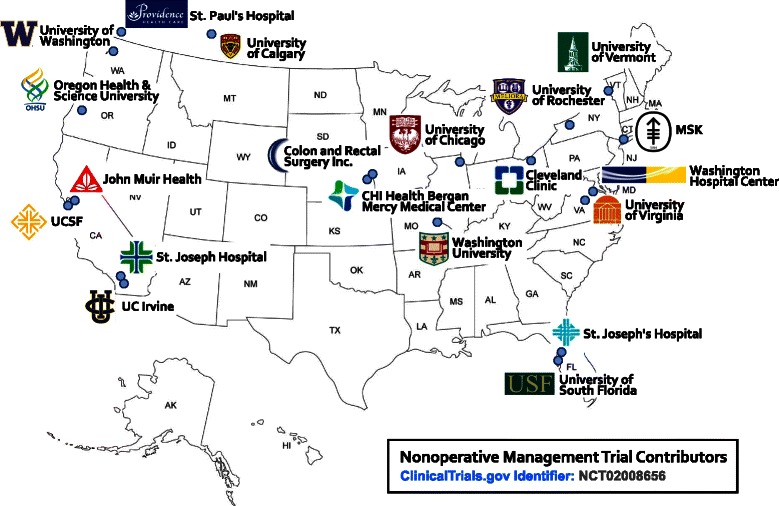
Nonoperative management trial contributors. A map of the United States is shown to demonstrate the multiple institutions involved in this described Phase II trial determining the feasibility of incorporating a NOM approach to the multimodality treatment of LARC patients

The majority of the rectal cancer consortium members (Fig. 2) were actively involved in a previous multicenter R01 NCI-funded project (1R01 CA090559-07): “The Timing of Rectal Cancer Response to Chemoradiation” (TIMING Trial; NIH NCT00335816; see recent work in *The Lancet Oncology*, PMID: 26187751) [9]. Each of these sites has been actively involved in the study, and each site has a proven ability to accrue patients and treat them according to the protocol.

### Coordination and monitoring

Memorial Sloan Kettering Cancer Center (MSKCC) is responsible for overseeing all fiscal and administrative arrangements with the consortium. The MSKCC Office of Clinical Research includes a Multicenter Protocol Group that provides regulatory and administrative support, and a Clinical Research Quality Assurance group responsible for auditing and monitoring investigator-initiated protocols. Participating sites will be monitored on an ongoing basis to ensure good data management, HIPAA compliance, and appropriate use of funds, and to address any local administrative challenges that may hinder recruitment. Monthly conference calls are instituted to discuss administrative and clinical updates. A monthly project update will be distributed to all Principal Investigators, Coordinators, and project affiliates. In addition, individual site training and new staff orientations will be offered throughout the year. For quality control purposes, an annual review will be performed to assess each site with respect to matters of recruitment, fiscal management, and data quality. Low-accruing sites will be evaluated to ensure that they have adequate staff, clear communication channels, and a screening and recruitment plan in place.

### Ethics, informed consent and safety

The Institutional Review Board (IRB) of MSKCC approved the final protocol, and the appropriate approvals have been obtained by each participating site. Physicians qualified to conduct the informed consent process must be certified in the protection of human subjects for research. Before protocol-specified procedures are carried out, investigators or their staffs will explain to patients the full details of the protocol and study procedures, as well as the risks involved, prior to their inclusion in the trial. Patients will also be informed that they are free to withdraw from the study at any time. All patients must sign an IRB-approved consent form indicating their consent to participate. This consent form will meet the requirements of the Code of Federal Regulations and the Institutional Review Boards of all participating centers. The consent form includes the nature and objectives, potential toxicities and benefits, of the intended study; the required length of therapy and the likely follow-up; alternatives to the proposed therapy (including available standard and investigational therapies); supportive care; the name(s) of the investigator(s) responsible for the protocol; the right of the patient to accept or refuse treatment, or to withdraw from the study.

Patient safety is of utmost importance. To ensure that patients are not put at undue risk, stopping guidelines and vigilant monitoring practices are in place. Safety will be assessed through documentation of adverse events during TNT and after surgery. Adverse events will be graded according to NCI CTCAE version 4, and surgical complications will be graded according to the Clavien-Dindo Classification [13]. Serious adverse events (SAEs) will be reported to the Institutional Review Board and recorded electronically.

To protect patients treated non-operatively against the risk of local tumor progression during follow-up, we will monitor continuously for resection with positive margins (R1 resection) based on repeated significance level. To protect patients against the risk of tumor progression, we will conduct an interim evaluation with clinical exam and radiology studies during neoadjuvant treatment. Patients with diagnosis of tumor progression during treatment will be removed from the study and treated according to standard of care. These cases will be reviewed by the Data and Safety Monitoring Committee. We will regularly monitor for adverse events and surgical complications. The results will be reviewed periodically by the Data and Safety Monitoring Committee to protect against the risk of increased adverse events and surgical complications.

The Principle Investigator (PI), Co-Investigators, research manager (KA), research project coordinator, and research service associate will be responsible for ensuring that all consent forms and electronic data gathered during the course of the study are stored in locked cabinets, a secure database, or password-protected HIPAA-approved electronic files. This applies to all consortium sites, and the MSKCC PI will be responsible for overseeing compliance. Remote monitoring and auditing will be performed on all consortium sites to ensure compliance. Clinical information will be entered into a HIPAA-compliant, confidential, password-protected clinical database at MSKCC, which only the PI, research manager, research project coordinator, and research service associate will be able to access. The research records will not be included in the patient’s hospital medical record.

### Statistical analyses in the context of the Specific Aims

The goal of Specific Aim 1 is to evaluate 3-year RFS in patients managed with INCT or CNCT, CRT and selective NOM, compared with standard historical controls treated with CRT and TME followed by ACT. We hypothesize that 85 % of patients with LARC treated with TNT and selective NOM will be alive and free of disease at 3 years. The 3-year disease-free survival (DFS) for similar patients (stage II/III LARC within 6 cm from the anal verge) treated according to the standard of care (CRT, TME, and ACT) is 75 % [10]. In this trial, each arm is designed as a single-stage study that discriminates between 3-year DFS rates of 75 % (historical control) and 85 % (study groups). For our power calculation, we assume uniform accrual over time and an exponential distribution for time to death. Using the approach proposed by Lawless (1982) [14], for 80 % power, a type I error of 5 %, and a one-sided test, we will require 101 patients per arm, accrued over a 4-year period, with an additional 3 years of follow-up. With a sample size of 101 patients, we will consider the trial worthy of further study if the 3-year DFS rate exceeds .82 (the upper critical value). We anticipate about 10 % loss to follow-up, and will recruit an additional 10 patients per arm to account for this. Patient accrual is expected to take 4 years, with approximately 5 patients accrued per month.

The goal of Specific Aim 2 is to compare outcomes in patients treated on the two arms of this study, with respect to rates of organ preservation, compliance with the neoadjuvant protocol, and adverse events. If both arms meet the endpoint in Specific Aim 1, we plan to use NOM rate to determine the more promising regimen using a “pick the winner” strategy. We will calculate the proportion of patients treated with NOM who are alive and free of disease 3 years following the start of the study. We will require at least 20 NOM patients in each arm to employ the following strategy: If there is a difference of 5 NOM patients between arms, the arm with more NOM patients will be deemed the winner. With 101 patients in each arm, 20 % NOM in patients treated with INCT and 30 % in patients treated with CNCT, we will have an 83 % probability of selecting CNCT, a 1 % probability of selecting INCT, and a 16 % probability of considering the study inconclusive. In addition, we will calculate therapy compliance using the following measures: number of days RT was held, the number of RT delays of ≥ 1 week, the number of dose delays and number of dose reductions in INCT and CNCT. We will also calculate the rate of grade 3 or higher adverse events and surgical complications in each treatment arm.

In regard to Specific Aim 3, we will measure patient-reported functional outcomes and QoL in patients with LARC treated with NOM, compared to patients treated with TME. The primary endpoint of QoL will be assessed using the EQ-5D index, an overall measure of QoL and health ranging from 0 (worst health) to 1 (best health). Comparison will be done using the two-sample t-test. In secondary analyses we will use a paired t-test to compare the EQ-5D index, measured at 1 year, in patients with durable NOM vs. TME. The differences in EQ-5D index from 1 year to 3 years will also be compared in patients with durable NOM vs. TME, using an ANCOVA model.

The goal of Specific Aim 4 is to investigate the diagnostic performance of conventional MRI and DW-MRI in identifying LARC patients treated with TNT who will benefit from NOM. This will be the first study investigating the diagnostic performance of conventional and DW-MRI in LARC patients treated with TNT. Therefore, this aim will be considered exploratory.

### Patient selection and eligibility criteria

In order to participate, patients must have not received previous chemotherapy, pelvic radiotherapy, or surgery for their rectal cancer. Specific inclusion and exclusion criteria are provided in Table 1. Biopsy confirmation of the primary rectal tumor by pathology must be performed at the participating institution before initiation of therapy. Patients who choose to participate will provide written informed consent prior to study enrollment. Patients will be recruited and consented in the Surgical Colorectal clinics, Medical Oncology clinics and Radiation Oncology clinics of participating institutions. Patients must undergo surgical, medical oncology and radiation oncology evaluation at each participating site. At baseline, each patient must undergo a full medical history and physical examination, including assessment of body weight, height, calculated body surface area and sitting vital signs (blood pressure, heart rate, respiratory rate, and temperature). Laboratory assessments include: a) CBC with differential, comprehensive biochemical screening profile (electrolytes, BUN, creatinine, AST, ALT, total bilirubin, total protein, albumin, alkaline phosphatase and glucose); b) CEA; and c) urinalysis. Patients will undergo baseline flexible sigmoidoscopy with a photograph of the tumor, as well as biopsy, proctoscopy, ERUS and MRI of the pelvis, to accurately assess the extent of the tumor. A requirement for study entry is rectal cancer, clinically characterized by evaluation with MRI as Stage II (T3-4, N0) or Stage III (any T, N1-2) . For females of childbearing potential, a serum pregnancy test is also required. Additional evaluations include baseline EKG and a signed informed consent. CT scan of the chest and abdomen is required to rule out disseminated metastatic disease. A PET scan will be performed to assist in planning radiotherapy.

**Table 1:** Inclusion and exclusion criteria for the trial

	Inclusion Criteria	Exclusion Criteria
General	•Histologically confirmed diagnosis of adenocarcinoma of the rectum •Patients must have Stage II (uT3, uN0) or Stage III (T1-3, N1-3) tum or, staged by MRI, that is potentially resectable •Locally advanced rectal cancer a menable to total mesorectal excision •No evidence of distant metastases ·No prior pelvic radiation therapy •No prior chemotherapy or surgery for rectal cancer •Age >18 years •No infections requiring systemic antibiotic treatment •Karnofsky >60% or ECOG 0-2 ·ANC > 1.5 cell/mm3, Hgb>8.0 gm/ dL, PLT>150,000/mm3, total bilirubin < or equal to 1.5 x upper limit of normal, AST > or equal to upper limit of normal, ALT< or equal to three times upper limit of normal	•Recurrent rectal cancer•Primary unresectable rectal cancer is defined as a primary rectal tumor which, on the basis of either physical examination or pelvic MRI, is deemed tobe adherent or fixed to adjacent pelvic structures. (en bloc resection will not achieve negative margins)•Serum creatinine level greater than 1.5 times the upper limit of normal•Patients who have received prior pelvic radiotherapy•Patients with a history of any arterial thrombotic event within the past 6 months, including angina (stable or unstable), MI, or CVA•Patients with any other concurrent medical or psychiatric condition or disease which, in the investigator's judgment, would make them inappropriate candidates for entry into this study•Patients with a history of a prior malignancy within the past 5 years, except for adequately treated basal cell or squamous cell skin cancer, or in situ cervical cancer. •Patients with a history of thrombotic episodes, such as deep venous thrombosis, pulmonary embolus, MI, or CVA occurring more than 6 months prior to enrollment may be considered for protocol participation, provided they are on stable doses of anticoagulant therapy. Patients who are anticoagulated for atrial fibrillation or other conditions may participate, provided they are on stable doses of anticoagulant therapy.• Patients receiving other anticancer or experimental therapy. No other experimental therapies (including chemotherapy, radiation, hormonal treatment, antibody therapy, immunotherapy, gene therapy, vaccine therapy, angiogenesis inhibitors, matrix metalloprotease inhibitors, thalidomide, anti-VEGF/Flk-1 monoclonal antibody, or other experimental drugs) of any kind are permitted while the patient is receiving study treatment.
Consent	Patients must read, agree to, and sign a statement of Informed Consent prior to participation in this study. MSKCC patients who do not read or understand English are eligible, but must have the consent form read to them in its entirety by an official translator, either from MSKCC or AT&T. Informed consent for non-literate or non-English speaking patients may not be obtained by using a relative or a member of the patient’s clinical team as a translator. Consortium sites must follow federal, local, and institutional regulations to ensure that non-English speaking patients are consented appropriately.	n/a
Women	•Women with childbearing potential who are negative for pregnancy test (urine or blood) and who agree to use effective contraceptive methods. A woman of childbearing potential is defined by one who is biologically capable of becoming pregnant. Reliable contraception should be used from trial screening and must be continued throughout the study.	Women who are pregnant or breastfeeding Women of childbearing potential who are unwilling or unable to use an acceptable method of birth control to avoid pregnancy for the entire study period, and for up to 4 weeks after the study.
Men	Male subjects must also agree to use effective contraception.	n/a

### Histopathology and biospecimens

We will collect tumor and normal tissue samples at baseline and during surgery in patients undergoing a TME. We will also collect blood at baseline and at different time points during and after treatment, to measure circulating DNA and miRNA. Pathologic assessment of the surgical specimens will be performed according to the College of American Pathologists guidelines. Completeness of the mesorectal excision, tumor regression grade (TRG), and tumor staging will be categorized according to the criteria specified in the 7th Edition of the AJCC Staging Manual [15].

### Neoadjuvant treatment

The neoadjuvant chemotherapy regimen is prescribed specifically as 8 cycles of FOLFOX or 6 cycles of CapeOX over a period of approximately 16 weeks. The CRT regimen consists of the standard MSK algorithms: a total of 5600 cGy of radiation (4500 cGy to the pelvis, with an integrated boost to the primary tumor and involved nodes of 5000 cGy, followed by a 600 cGy boost to the primary tumor and involved nodes) in 28 fractions of 180-200 cGy each, over a 5-6-week period. Starting on the first day of RT, patients receive 5-FU administered by continuous infusion, or capecitabine, for the duration of radiotherapy.

### Evaluation of response and resectability

**Fig. 3:**
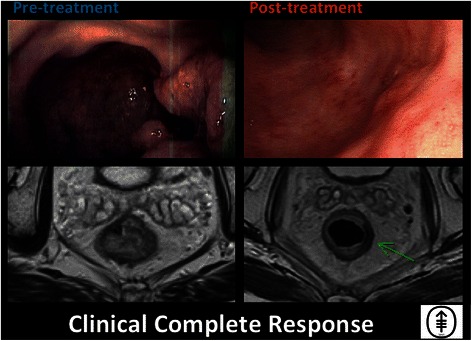
Clinical complete response. Endoscopic and T2-weighted MRI images, both pre- and post-treatment, are shown for a patient who has achieved a clinical complete response. Images displayed were taken from endoscopic and MRI views of an 85 year-old man who underwent capecitabine CRT followed by consolidation chemotherapy with CapeOx and was determined to achieve cCR both clinically and radiologically. In the post-treatment T2-weighted MRI image shown, the green arrow points to the prior site of the tumor

**Fig. 4:**
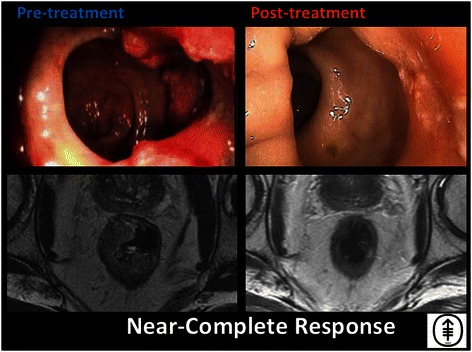
Near-complete response. Endoscopic and T2-weighted MRI images both pre- and post-treatment are shown for a patient who has achieved a near complete response. This is a 74-year-old man who underwent 8 cycles of induction FOLFOX followed by CRT, and achieved a near-cCR. A biopsy obtained in surveillance was determined to contain residual cancer; therefore, the patient was referred for TME

**Fig. 5:**
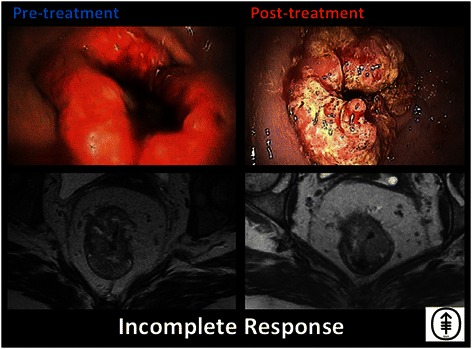
. Incomplete response. Endoscopic and T2-weighted MRI images both pre- and post-treatment are shown for a patient who experienced no significant response to induction chemotherapy followed by CRT. This is a 45-year-old woman who underwent 8 cycles of induction FOLFOX followed by CRT with minimal or no response. The patient was therefore referred for TME

An interval evaluation will be conducted after completion of INCT in Arm 1, and after completion of CRT in Arm 2 (Fig. 1). Patients in both arms will be re-staged after completion of all neoadjuvant therapy. Patients with cCR or near-complete clinical response (Figs. 3 and 4) will be treated with NOM. Patients treated with NOM will be followed every 3 months for 2 years, and every 6 months thereafter. Patients with an incomplete response and residual tumor at the primary site will undergo a TME (see Fig. 5). Patients treated with TME will be followed according to NCCN guidelines [16,17].

### Guidelines for tumor measurement

For the endoscopic exam, the length of the tumor is defined as the difference between the distance of the proximal and distal margins in relation to the anal verge. For MRI/CT, the standard and DW-MRI sequences will be obtained in 1.5 T or 3 T units by using a phased-array body coil. All imaging studies will be interpreted by expert radiology staff at the patient’s primary treatment center to determine patient eligibility, clinical staging, and tumor response, according to standard clinical criteria.

### Criteria for response after neoadjuvant therapy

The primary tumor and the regional lymph nodes will be evaluated and measured by endoscopic exam and rectal MRI during re-staging. Central imaging review will be performed by the Radiology PI at MSKCC after receipt of baseline and post-TNT images. For quality control purposes, baseline MRI images will be collected for the first two patients enrolled at each site. MRI images taken at interval evaluation are required for all MSKCC patients, and are recommended for participating sites. These interval evaluation images will be submitted on disc for central review. All submitted discs will be de-identified, labeled using the unique study number, and in the DICOM format. MRI images taken at re-staging will be collected for all patients, and sent to MSKCC on disc for central review. Local and central interpretation will be tracked. Discrepancies between clinical examination and imaging will be resolved by the local investigator, but will be communicated to the PI and Radiology PI at MSKCC. In the event that the radiologist at MSKCC disagrees with a site assessment, these discrepancies will be relayed to local the PI and radiologist. No response will be relayed to the participating site if, upon review of submitted images, there are no discrepancies in assessment. In general, clinical examination will prevail over imaging in assessment of local tumor response. Our regression schema, an online resource we have created for all investigators, and the tabulated form will be used to enhance uniformity of evaluation and provide quantitative endpoints which we can use to provide a more precise, consistent means of evaluating assessment at the end of the study. Details of this three-tiered response regression schema can be found below (Table 2, and see below).

**Table 2:** Memorial Sloan Kettering Regression Schema

	Complete Response	Near Complete Response	Incomplete Response
Endoscopy	Flat, white scar Telangiectasia No ulcer No nodularity	Irregular mucosa Small mucosal nodules or minor mucosal abnormality Superficial ulceration Mild persisting erythema of the scar	Visible tumor
Digital Rectal Exam	Normal	Smooth induration or minor mucosal abnormalities	Palpable tumor nodules
MRI-T2W	Only dark T2 signal, no intermediate T2 signal	Mostly dark T2 signal, some remaining intermediate signal	More intermediate than dark T2 signal, no T2 scar
	AND	AND/OR	AND/OR
	No visible lymph nodes	Partial regression of lymph nodes	No regression of lymph nodes
MRI-DW	No visible tumor on B800-B1000 signal	Significant regression of signal on B800-B1000	Insignificant regression of signal on B800-B1000
	AND/OR	AND/OR	AND/OR
	Lack of or low signal on ADC map Uniform, linear signal in wall above tumor is ok	Minimal or low residual signal on ADC map	Obvious low signal on ADC map

### Treatment decisions after completion of TNT: The Memorial Sloan Kettering Regression Schema

One of the main challenges to implementation of a NOM protocol at a multi-institutional level is the development of uniform and reproducible criteria for tumor response. To that end, our consortium organized a multidisciplinary videoconference on 26 January 2014 aimed at developing a consensus on the clinical criteria of tumor response. The participants—colorectal surgeons, medical oncologists, radiation oncologists, pathologists and radiologists—elaborated a three-tiered assessment of response/regression schema to differentiate between patients with a cCR who are therefore candidates for NOM, from those without a cCR who are candidates for TME. This regression schema was further discussed at the American Society of Colon and Rectal Surgeons Annual Scientific Meeting in Hollywood, Florida in May 2014, and finalized at the 2014 American College of Surgeons Clinical Congress in October 2014. The regression schema is presented in Table 2, and will be available online by requesting access via Dr. Smith (smithj5@mskcc.org). The regression schema is based on relatively subjective endoscopic and radiological criteria and has not been validated yet; however, it may provide some degree of uniformity that may help to maintain consistency and reduce variability between institutions.

## Discussion

This study represents an attempt to investigate, in a multi-institutional setting, the feasibility of rectal preservation in a number of LARC patients who develop a cCR after a novel neoadjuvant therapy protocol. It has the potential to change the treatment paradigm for many patients with rectal cancer. The study design is innovative because it compares 3-year DFS in a group of LARC patients treated according to a protocol incorporating TNT and NOM, to a similar group of historical controls treated according to the standard CRT, TME and ACT protocol. The studies published thus far on the safety of NOM in LARC patients have compared the survival of a selected group of patients with a cCR and NOM, to a selected group of patients with a pCR after TME. While these reports provide an estimate of the safety of NOM, they do not provide accurate information on the impact of selective use of NOM on outcomes in the entire LARC population. Our study will be the first to determine the proportion of patients with LARC who will be candidates for NOM, and the impact of NOM on 3-year DFS in the entire LARC population.

There is now conclusive evidence that tumor response to chemotherapy and radiation requires time, and that some tumors achieve a maximal response only after several months. However, in the studies on NOM published to date, tumor response was assessed a few weeks after completing the standard neoadjuvant protocol. It is possible that many patients who were considered non-responders early on, and were therefore not offered NOM, might actually have been complete responders given a longer observation period. In our study, assessment of tumor response is done only after all neoadjuvant treatment is delivered. In patients completing the entire treatment, final assessment of tumor response—and the decision between NOM and TME—will be done approximately 32 weeks after initiation of therapy. We think this will provide sufficient time for tumor response.

In the past, LR, likely secondary to inadequate surgery, was the most common form of relapse in patients with LARC. Advances in imaging, surgical technique, and adjuvant therapy have reduced the risk of LR dramatically. Nowadays patients with seemingly localized LARC who die of disease after undergoing treatment with curative intent, succumb to DM (which probably develops from occult micrometastasis present at the time of treatment of the primary tumor). Therefore, as in patients with stage III colon cancer, current guidelines recommend postoperative chemotherapy for patients with LARC. While chemotherapy seems to improve survival compared to no chemotherapy, it is not as effective as would be desired. Although this may be due, in part, to the fact that the agents used are not effective against every single cancer, it is also the case that many rectal cancer patients never start chemotherapy after surgery, and less than half complete the entire treatment [5,18]. In light of our previous experience with neoadjuvant therapy, in this study we will deliver all systemic chemotherapy before assessing tumor response. Delivering systemic chemotherapy in the neoadjuvant setting will not only improve compliance and address the problem of micrometastasis earlier, compared to the standard treatment algorithm of CRT, TME and ACT; it may also contribute to enhanced tumor response. As a result of lengthening the treatment time by administering NACT, this study will provide a closer estimate of the proportion of patients with LARC who will respond to chemotherapy and radiation, and thus be eligible for NOM.

There are some potential advantages to using NACT before or after CRT. Delivering NACT before CRT has the advantage of treating occult micrometastasis earlier. As the patients will be treatment naïve, compliance with systemic CT may be higher. However, NACT-related toxicity may potentially reduce compliance with CRT. On the other hand, starting treatment with CRT delays delivery of the full dose of systemic chemotherapy used to treat micrometastatic disease, and this may reduce compliance with NACT. An important question is which treatment arm will demonstrate greater tumor response. The results of the TIMING trial compared to the other studies suggest that pCR rates are higher with CRT and CNCT compared to INCT and CRT (see recent work in *The Lancet Oncology*:  PMID:  26187751). The differences between CRT and CNCT versus INCT and CRT may be due to patient selection or delay in the time to assessment of response, rather than treatment effect [9]. However, in patients with squamous cell carcinomas (of various locations) undergoing neoadjuvant therapy, there is evidence that longer duration of treatment, measured from the first day of any therapy to the last day of radiation, is associated with increased local failure and/or decreased survival [19]. In anal cancer patients, overall treatment time, but not radiotherapy time, is associated with a high rate of local failure, suggesting that induction chemotherapy may contribute to local failure by increasing the total treatment time [20]. Our study will test this hypothesis to assess whether the same phenomenon occurs in adenocarcinomas.

Patients with distal rectal cancer who require a CAA as part of their surgical treatment also have a temporary diverting loop ileostomy to reduce the risk of pelvic sepsis. Patients treated according to the standard protocol of CRT, TME and ACT must live with a diverting loop ileostomy for many months until they complete postoperative adjuvant chemotherapy. While the ileostomy is effective in reducing major pelvic infections, it is an inconvenience to the patient; it is also the source of complications, such as dehydration and electrolyte imbalances, which often require readmission to hospital [21]. Delivering all systemic chemotherapy in the neoadjuvant setting will shorten ileostomy time.

One of the main criticisms of NOM in patients with LARC is the poor correlation between cCR (based on both clinical examination and imaging studies) and pCR to neoadjuvant therapy. However, response has typically been assessed a few weeks after completion of neoadjuvant therapy, before some tumors have achieved maximal response, and before treatment-related tissue changes have had an opportunity to subside. This will be the first study to investigate the accuracy of clinical examination and imaging studies in assessing tumor response after a much longer treatment interval. It will also help validate a set of predefined criteria regarding clinical and radiological response(see Table 2).

Additionally, we will evaluate the feasibility of using circulating tumor DNA and miRNA profiles in plasma, to monitor tumor response to TNT, in rectal cancer patients treated in both of the protocol arms. Genomic analysis by next-generation sequencing will be done to profile rectal cancer treated with neoadjuvant chemotherapy and radiation. The separation of responders from non-responders will facilitate investigation of the molecular mechanisms of tumor resistance to neoadjuvant therapy through genomic analysis of rectal cancer before and after treatment. We think that this will lead to more precise treatments of individual rectal cancer patients based on the molecular profile associated with each individual tumor.

In summary, this study will not only explore the feasibility of NOM in a selected group of patients with LARC who respond to neoadjuvant therapy. It will also provide a wealth of information about the response of LARC to chemotherapy and radiation. By delivering all adjuvant therapy up front, we will be able to discriminate (or separate) responders from non-responders, determining the real proportion of patients who will benefit from NOM, facilitating the clinical and radiological identification of responders, and facilitating molecular profiling. Finally, the study will help determine the gains in QoL associated with organ preservation.

### Overview of Clinical Trial 13-213 and summary statements:

Herein we postulate that:

1. *Delivery of chemotherapy before rather than after surgery will improve survival, because it will treat microscopic tumor deposits several months earlier, will increase the proportion of patients completing the entire treatment, and may reduce the size of the primary tumor.* This study will compare the two different TNT regimens in two randomized treatment arms. One treatment arm will receive systemic chemotherapy before chemoradiation, and the other treatment arm will receive it after chemoradiation.
2. *Delivery of chemotherapy before rather than after surgery will shorten the amount of time before ileostomy reversal.* This study investigates the feasibility and efficacy of delivering all adjuvant treatment before surgery in LARC patients. Some previous phase II trials have investigated the use of chemotherapy before surgery in such patients, but the chemotherapy delivered in these trials was only a portion of that normally used after surgery. In this study, we deliver all chemotherapy before surgery (TNT). The results could be confirmed in a phase III trial, and may represent the groundwork for a paradigm shift in the treatment of rectal cancer.
3. *Avoiding surgery selectively in patients with tumors that respond to CRT will reduce over-treatment and improve quality of life.* The underlying theme here is individualized treatment, tailored in a more precise fashion. The potential gains for these patients in terms of improved QoL will be significant. Over 20,000 new stage II and III rectal cancers are diagnosed in the United States every year. Assuming a 30 % rate of complete response, this could mean that 6,000 patients will benefit from NOM, leading to significant economic benefits and better QoL.

#### Proposed value-added contribution of this trial

This is the first prospective study investigating the feasibility of NOM in rectal cancer patients who have a complete response to neoadjuvant therapy. Additionally, we will use the first regression schema to be prospectively validated in a multi-institutional setting for the assessment of response. We believe that the proposed treatment will improve survival and preserve QoL in LARC patients, in a more precise, patient-centric manner.

## Abbreviations

ADC: apparent diffusion coefficient
cCR: clinical complete response
CNCT: consolidation neoadjuvant chemotherapy
CRT: chemoradiation therapy
CT: adjuvant chemotherapy
DFS: disease-free survival
DM: distant metastases
INCT: induction neoadjuvant chemotherapy
LARC: locally advanced rectal cancer
NCT: neoadjuvant chemotherapy
NOM: non-operative management
pCR: pathological complete response
PFS: progression-free survival
ROI: region of interest
TME: total mesorectal excision
TNT: total neoadjuvant therapy
